# Evaluation of a Contactless Sleep Monitoring Device for Sleep Stage Detection at Home in a Healthy Population: Prospective Study in Free-Living Conditions

**DOI:** 10.2196/77033

**Published:** 2026-04-02

**Authors:** Marie-Ange Stefanos, Guillaume de Laboulaye, David Campo, Martin de Gourcuff, Pierre Escourrou, Boris Matrot, Anna Sigridur Islind, Pierre Alexis Geoffroy

**Affiliations:** 1 Inserm, F-75019, NeuroDiderot, SleepCmd, Université Paris Cité, Paris, France; 2 Department of Computer Science, Reykjavík University, Reykjavik, Iceland; 3 Withings, 2 rue Maurice Hartmann, Issy-les-Moulineaux, 92130, France, 33 141460460; 4 Centre Interdisciplinaire du Sommeil, Paris, France; 5 Département de Psychiatrie et D’addictologie, Hopital Bichat - Claude Bernard, F-75018, AP-HP, GHU Paris Nord, DMU Neurosciences, Paris, France; 6 GHU Paris - Psychiatry and Neurosciences, Centre ChronoS, Paris, France

**Keywords:** health, informatics, nearable, wearable, sleep stage, home polysomnography, performance assessment, healthy population, sleep monitoring device

## Abstract

**Background:**

Sleep is essential for overall health and well-being, but assessing sleep architecture is often costly and time-consuming, relying primarily on polysomnography (PSG). While wrist-worn wearables offer alternatives, they face limitations regarding user compliance, such as battery charging and physical discomfort. Nearable devices address these burdens, but they regularly lack rigorous validation, especially in real-world settings.

**Objective:**

This study evaluates the accuracy and reliability of the Withings Sleep Analyzer (WSA), a contactless sleep monitoring device, compared to PSG in a home setting using a large and diverse cohort of healthy individuals.

**Methods:**

A total of 117 healthy volunteers (69 women; mean 39.9, SD 11.4 years), prospectively recruited from the general population, underwent home-based PSG and simultaneous WSA recording. The study was conducted under free-living conditions, without constraints on substance intake, prebedtime activity, or forced sleep schedules. The main outcomes were the device’s performance in sleep-wake distinction and sleep stage identification using accuracy, kappa, sensitivity, specificity, and the mean absolute error of sleep measures on the entire population and demographic, clinical, and environmental subgroups.

**Results:**

WSA demonstrates high sensitivity (93%, 95% CI 92%-94%) for sleep detection and moderate sensitivity (73%, 95% CI 69%-77%) for wakefulness, achieving an overall accuracy of 87% (95% CI 86%-87%) for sleep-wake distinction. The device showed consistent performance across various demographic subgroups, including different age, BMI, mattress, and sleep arrangements (with or without bed partner) categories. Challenges were noted in accurately classifying specific sleep stages, particularly in distinguishing between light and deep sleep, with a mean accuracy of 63% (95% CI 62%-65%) and a Cohen κ of 0.49 (95% CI 0.47-0.51). The WSA tended to overestimate total sleep time (20 min, 95% CI 10 min to 31 min) and light sleep (1 h 21 min, 95% CI 1 h 8 min to 1 h 36 min) while underestimating rapid eye movement (−15 min, 95% CI −23 min to −8 min) and deep sleep (−46 min, 95% CI −59 min to −34 min) durations. Disagreements between expert reviewers were mirrored in part by the WSA’s misclassifications. Participants reported altered perceived sleep quality during the night with the PSG, suggesting discomfort during sleep.

**Conclusions:**

Being contactless and placed under the mattress, the WSA offers a promising approach to long-term sleep monitoring in natural home environments. It shows competitive performance in sleep-wake and sleep stage identification compared to other consumer devices. Progress in wearable and nearable devices is necessary to enhance their accuracy to better support the monitoring of populations with impaired sleep, although limited by an imperfect gold standard. This work also emphasizes the importance of using large, diverse, and challenging datasets, as well as the need for a standardized methodology for accurate sleep stage classification.

## Introduction

Sleep is an essential component of human health and plays an important role in daily functioning and overall well-being [1]. Poor sleep quality can significantly impact multiple aspects of life, including physical health, social interactions, and emotional regulation [2,3]. Moreover, poor sleep quality can diminish alertness and cognitive function, increasing the risk of accidents. Furthermore, inadequate sleep has been linked to numerous health issues, such as metabolic disorders, cardiovascular disease, neurocognitive impairments, and mental health challenges [4]. In severe cases, poor sleep quality can lead to critical outcomes, including an increased risk of suicide [5]. Despite the recognized importance of sleep, sleep-related difficulties remain common in the general population [6]. Approximately 30% of adults experience at least one symptom of nocturnal insomnia on a chronic basis [6], over 15% report sleepiness that affects daily activities [7], and more than 10% experience obstructive sleep apnea [8].

Polysomnography (PSG), considered the gold standard for identifying the micro and macro sleep architecture, records a variety of signals (electroencephalogram [EEG], electrooculogram [EOG], electromyogram [EMG], electrocardiogram [ECG], pulse oximetry, air flow, and breathing effort [9]). As the hypnogram helps physicians identify abnormal sleep patterns, quantify sleep fragmentation, and analyze sleep stage distribution, it supports the diagnosis of various sleep disorders, such as sleep-related breathing disorders, narcolepsy, hypersomnia, and disturbing sleep-related behaviors [10].

PSG has limitations. It often involves extensive setup time and significant costs, making it less feasible for routine monitoring [11]. It is scored manually, which is a labor-intensive process that can lead to long waiting times and has been shown to be prone to human error [12]. Additionally, since it is typically conducted over just a few nights, it fails to capture the full picture of an individual’s circadian rhythm and night-to-night variability [13,14]. In most cases, PSG is conducted in a sleep lab with numerous sensors that can disrupt natural sleep patterns [15]. For all these reasons, PSG results in data that may not accurately represent a person’s typical sleep in their own home [16].

Ambulatory PSG attempts to address these limitations. Lab studies support its accuracy [17-19]. However, in an uncontrolled home setting, sleep metrics may differ; for example, total sleep time (TST) can increase, potentially due to the absence of lab-induced constraints on waking time [20].

The need for more accessible and cost-effective solutions for multinight sleep monitoring remains, driven by the desire to enhance our understanding of sleep quality and facilitate the identification of potential sleep disorders [14,21]. Home-based sleep monitoring devices can enable continuous tracking in the comfort of one’s own home, while providing data that reflect real-life conditions more accurately than laboratory-based examinations [22]. This can provide a more realistic picture of an individual’s sleep patterns and helps to address the limitations of traditional sleep studies [23].

To address the challenges of cost and complexity in multinight assessments, wearable sleep devices are commonly used to detect sleep and wake states. These devices increasingly combine actigraphy, which tracks body movements [16], with photoplethysmography (PPG) sensors to increase the accuracy of sleep measures estimation [23]. These devices still show two limitations. First, they struggle to accurately differentiate between wakefulness and sleep when individuals remain still while awake [24]. Second, wearable devices require users to maintain proper charging and wearing, leading to frequent data loss and inconsistent performance [25]. In contrast, nearables, which are not in direct contact with the individual, eliminate the need for charging or wearing a device. Nearables for measuring sleep are typically positioned under the mattress or at the bedside and require minimal intervention after setup.

Withings Sleep Analyzer (WSA) is a nearable device designed to provide a noninvasive alternative for monitoring sleep stages over consecutive nights. Despite the proliferation of wearable and nearable sleep-tracking devices, there is a notable lack of rigorous evaluation of the algorithms, leading to concerns about data accuracy and reliability [26,27]. To date, the WSA performance to detect apnea has been evaluated in laboratory conditions [28-32], for which it is European Conformity (Conformité Européenne) marked and Food and Drug Administration–cleared. In addition, it has been assessed for nocturnal sleep periods detection [33] and cardiorespiratory monitoring [34]. However, validation of its sleep stage detection algorithm remains limited. This study, therefore, aims to assess the WSA sleep stage identification algorithm in a real-world home setting in a cohort of 117 healthy adult participants.

There are several important methodological issues addressed in this work. Existing research in sleep-tracking technologies highlights several persistent problems and limitations. Many studies, such as those on the WatchPAT [35] and Somnofy [36] devices, suggest potential utility but often lack comprehensive validation across diverse populations and real-world settings. Evaluations of consumer-grade devices, like those conducted by Chinoy et al [37] and Kainec et al [38], are frequently limited by small sample sizes and strict exclusion criteria (young individuals with controlled caffeine, alcohol, screen time, and sleep regularity), which limit the generalizability of their findings. These studies typically occur in controlled environments, such as sleep labs, which do not accurately reflect regular user conditions at home.

Moreover, the assessment of device accuracy in these studies is often complicated by two distinct factors. First, the absence of synchronized epoch-by-epoch data in some reports limits performance evaluation to night-scale sleep measures, such as TST, precluding the calculation of detailed classification metrics (eg, accuracy, sensitivity, and specificity). Second, the practice of using lights-off and lights-on times to define the temporal analysis window, which excludes all data outside this interval, impairs the reliability of the computed error for metrics like sleep onset latency. This method indeed assumes observable in-bed time is equivalent to the intention to initiate sleep, which cannot be passively identified in free-living conditions [39].

Recent publications emphasize the importance of assessing these devices under everyday conditions, aligning with our work (eg, [40]). Other research emphasizes the need for standardized evaluation protocols and methodological consistency across studies, reinforcing the approach taken in our research [41-43]. These studies collectively highlight the critical need for standardized and rigorous evaluation methods in sleep monitoring technology. Benchmarking efforts of devices are also being addressed, as recent studies manifest [37,38,44-47].

Our study contributes to the growing literature on sleep tracking by assessing the WSA’s performance in a home setting, on a large and diverse sample of healthy individuals, in free-living conditions, that is, without constraints about substance intake, activity before bed, or forced sleeping period, providing insights into the device’s accuracy and reliability in real-world conditions. The primary goal is to evaluate WSA’s performance using classification metrics and mean absolute error (MAE) on standard sleep metrics [48]. Additionally, we compare performance across subgroups based on demographic and clinical characteristics, such as sex, age, BMI, bed partner presence, mattress type, but also Pittsburgh Sleep Quality Index (PSQI) score [49], mattress, and thickness.

By addressing these gaps, our study enhances the understanding of WSA’s capabilities and contributes to the development of more reliable and user-friendly sleep-tracking devices.

## Methods

### Ethical Considerations

Data were collected during a clinical study approved by the French Institutional Review Board (approval number: 2018-A03129-46) and conducted by Withings from 2020 to 2022. The inclusion process for the study was conducted prospectively by a third party, a general-purpose survey company owning listings of self-declared individuals from the general population. Participants were contacted based on the inclusion and exclusion criteria. In particular, the contacted persons had to have no preexisting medical conditions that would necessitate a PSG examination, and inclusion criteria aimed to ensure a balanced distribution of BMI within each age group. The cohort consisted of a consecutive series of 194 volunteers who provided informed consent. To guarantee data privacy, a unique, randomized anonymization code was assigned to each participant (used for both WSA and PSG data correspondence). Withings only had access to these pseudonymized datasets and never had access to direct identifiers. No compensation from Withings was provided to the participants.

### Dataset

#### Participants

A total of 194 participants slept for the first night at home with WSA and the CID-LXe portable PSG (CIDELEC), both installed by a technician the evening before the recording. The night after that, the participants slept at home with the WSA, without the PSG. Each participant was asked to answer once to the PSQI questionnaire upon enrollment. After each night, participants were asked to complete a survey addressing the following: their sleep quality for that night, a comparison of that night’s sleep quality to their usual sleep, whether they took sleeping pills, and any sleep disturbances related to the sensors.

**Figure 1.**
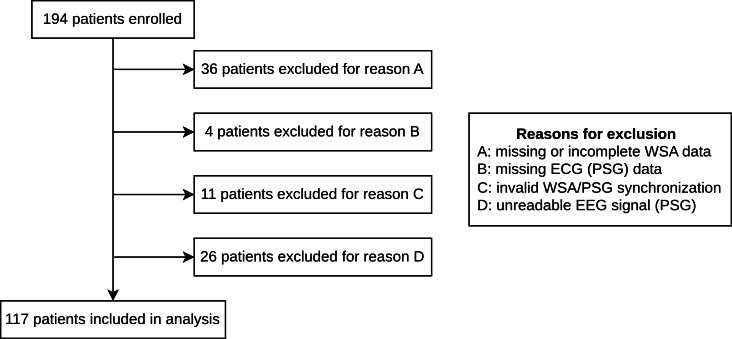
Study inclusions flowchart. ECG: electrocardiogram; EEG: electroencephalogram; PSG: polysomnography; WSA: Withings Sleep Analyzer.

As illustrated in the flowchart (Figure 1), several sources of data loss have led to the inclusion of 117 participants in this analysis. This trial was notably affected by data collection issues due to a dedicated firmware meant to collect high-frequency sensor data (reason for exclusion A). Unlike the commercial version which transmits aggregated data, the devices in this study were programmed to upload raw, high-frequency sensor signals, which introduced connectivity issues detailed in the Limitations section. We performed Little’s missing completely at random (MCAR) test, which did not reject the null hypothesis of MCAR data due to reasons A, C, and D (*P*>.05). While a statistically significant result was observed for reason B (*P*=.04), this subgroup comprised only 4 participants and was therefore unlikely to represent a source of systematic bias (see Section A of Multimedia Appendix 1 for detailed results).

#### Procedure and Study Design

WSA sampled the raw data from the pressure sensors at 250 Hz and preprocessed data from the microphone at 8 Hz. The reference device was CID-LXe PSG (CIDELEC) that recorded the following signals: 3 EEG, 1 ECG, 2 EOG, 1 submental EMG, and ambient light. Based on experience from past investigations, the digital oximeter and the nasal cannula are among the most sleep-disruptive sensors during PSG, even in a home setting. To minimize this sleep disruption and obtain the most natural possible sleep, PSG was therefore done at home with only EEG, EMG, ECG, and ambient light sensors. As sleep stage annotations from expert reviewers are based on EEG and light sensors only [50], the gold standard reference quality is not affected by the removal of the oximeter and the nasal cannula.

### Data Processing

**Figure 2.**
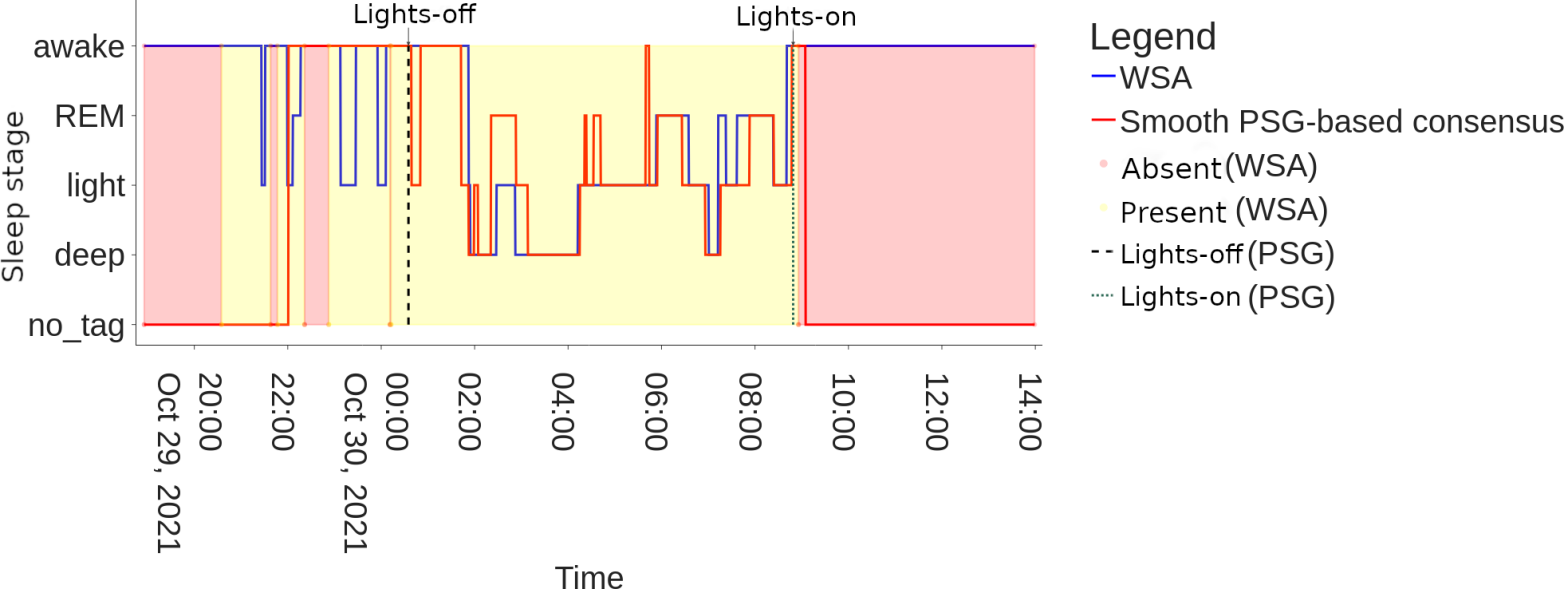
Example of hypnogram (participant 20211029_1) showing WSA sleep stages and the reference (PSG consensus). PSG: polysomnography; REM: rapid eye movement (sleep stage); WSA: Withings Sleep Analyzer.

WSA is designed to identify sleep stages over 6-minute windows. As a result, this analysis focuses on evaluating WSA’s performance in detecting stages longer than 6 minutes. It should be kept in mind that sleep stages shorter than this are ignored by the device in this study. In addition, WSA does not distinguish stages N1 and N2, so for the aim of comparison, the consensus sleep stages N1 and N2 obtained from the PSG were combined into the “light” category. Rapid eye movement (REM) and awake stages of the WSA and the consensus are directly comparable, and N3 is compared with the stage “deep sleep” from the WSA. The resulting stages are illustrated in Figure 2.

Two independent certified sleep technologists annotated the PSG signals in 30-second epochs, classifying sleep stages as N1, N2, N3, REM, and awake, following the American Academy of Sleep Medicine (AASM) scoring manual [48]. A third professional reviewed the 2 initial readings and established a consensus for reference data. Sleep stage comparisons were performed over windows of 30 seconds. To evaluate WSA’s performance in detecting sleep stages longer than 6 minutes, consensus data were smoothed by selecting for each 6-minute window the stage with the highest occurrence of 30-second epochs. For more details about the procedure, please refer to Section A of Multimedia Appendix 1.

In addition to standard sleep quality measures calculated over the night, such as TST and the duration of each stage, evaluating stage classification required synchronizing epoch-by-epoch data from both devices over time. Synchronization of the clocks of the devices was obtained by maximizing the correlation of the breathing signals of the two devices [51]. To verify the robustness of our breathing-based synchronization, we assessed the impact of temporal shifts (up to ±90 seconds) on classification performance [39,52]. This sensitivity analysis revealed negligible variations, with the SD for sensitivity and specificity across all sleep stages remaining below 0.005. Details of the procedure can be found in Section A of Multimedia Appendix 1.

An example hypnogram is presented in Figure 2, showing the smoothed, PSG-based consensus in red and WSA sleep stages in blue. WSA indicates bed absence/presence, marked respectively by a red and yellow background, information which is absent from the PSG data. The consensus annotations use instead the events lights-on/off detected by the ambient light sensor of the PSG as proxies for bed-in and bed-out events. For further information on challenges arising from using lights-off/on timestamps as proxies for bed presence, please refer to Section A of Multimedia Appendix 1.

### Performance Evaluation Metrics and Tools

Standard sleep quality measures used for this analysis and based on the AASM scoring manual [48] are the following: total sleep time, time spent in each sleep stage (TIREM, TILight, TIDeep, and TIWake), proportion of the time spent in each sleep stage compared to total sleep time (PIREM, PILight, PIDeep, and PIWake), and number of episodes in each sleep stage (NEREM, NELight, NEDeep, and NEWake) [50]. Each of these measures has been computed by each device (PSG and WSA) and then compared using the MAE and a Bland-Altman analysis, described in Section B of Multimedia Appendix 1.

Epoch-by-epoch agreement is assessed using standard classification metrics. Subgroup analyses were conducted to determine whether WSA performance is affected by demographic, clinical, and environmental factors. Details regarding the evaluation metrics and subgroup analyses are available in Sections B and C of Multimedia Appendix 1.

To compare the median MAE between different groups (categories of a given metadata as listed in Table S3 in Multimedia Appendix 1), a Kruskal-Wallis test has been performed on the MAE obtained on all the metadata described in Table S3 in Multimedia Appendix 1 for each of the following sleep quality measures (TST, TIREM, TILight, TIDeep, PIREM, PILight, and PIDeep) and for the number of episodes in each sleep stage.

To compare the MAE variance between different groups, a Brown-Forsythe test has been performed on the same data for the same sleep quality measures. The Holm-Bonferroni correction is used after both statistical tests to address the issue of multiple comparisons.

## Results

### Patient Characteristics

Demographic and clinical data are summarized in Table 1 for the population included in the analyses.

**Table 1.** Demographic characteristics and clinical data for the entire population (N=117).

Characteristics	Values
Demographics	
Age (years)	
Mean (SD)	39.9 (11.4)
Min-max	19‐69
Height (cm)	
Mean (SD)	171.6 (8.3)
Min-max	153‐194
Weight (kg)	
Mean (SD)	70.4 (12.0)
Min-max	49‐103
BMI (kg/m²)	
Mean (SD)	23.9 (3.3)
Min-max	17.9‐30.9
Sex, n (%)	
Male	48 (41)
Female	69 (59)
Skin type^a^, n (%)	
Skin type 1	85 (75)
Skin type 2	21 (18)
Skin type 3	8 (7)
Known pathologies, n (%)	
Asthma	5 (4)
Arterial hypertension	3 (<3)
Bipolar disorder	1 (<1)
Ulcerative colitis	1 (<1)
Ankylosing spondylitis	1 (<1)
Pathologies, n (%)	
Cardiovascular^b^	4 (4)
Neurological^c^	0 (0)
Digestive^d^	0 (0)
Metabolic^e^	2 (2)
Psychiatric^f^	0 (0)
Other	6 (5)
PSQI^g^ questionnaire [49]	
PSQI score	
Mean (SD)	6.0 (2.9)
Min-max	0‐15
Component 1: subjective sleep quality	
Mean (SD)	1.2 (0.7)
Min-max	0‐3
Component 2: sleep latency	
Mean (SD)	1.3 (0.9)
Min-max	0‐3
Component 3: sleep duration	
Mean (SD)	0.8 (0.8)
Min-max	0‐2
Component 4: habitual sleep efficiency	
Mean (SD)	0.4 (0.7)
Min-max	0‐3
Component 5: sleep disturbances	
Mean (SD)	1.3 (0.5)
Min-max	0‐3
Component 6: use of sleeping medications	
Mean (SD)	0.2 (0.6)
Min-max	0‐3
Component 7: daytime dysfunction	
Mean (SD)	0.9 (0.8)
Min-max	0‐3
Medications, n (%)	
Antihypertensive	3 (3)
Antidepressant	2 (2)
Hypnotics	1 (1)
Corticoid	1 (1)
Antiepileptic	1 (1)
Antipsychotic	1 (1)
Mattress type, n (%)	
Foam	41 (35)
Spring	29 (25)
Latex	24 (21)
Memory foam	12 (10)
Other	8 (7)
Unknown	3 (3)
Bed partner, n (%)	
Yes	64 (55)
No	52 (45)
Sensors disturbance (due to PSG^h^ device), n (%)	
Yes	46 (39)
No	71 (61)

a Skin type is based on the reduced Fitzpatrick scale.

b Arterial hypertension, atrial fibrillation, or myocardial infarction.

c Parkinson, stroke, adenoma, manic-depression, or epilepsy.

d Hemolytic anemia due to glutathione reductase deficiency, ulcer, sleeve, or diverticula.

e Diabetes, hypothyroidism, dyslipidemia, or obesity/overweight.

f Depression, insomnia, or anxiety.

g PSQI: Pittsburgh Sleep Quality Index.

h PSG: polysomnography.

A total of 117, mostly healthy, participants were included in the analysis. The cohort comprised 59% (n=69) women and 41% (n=48) men, with a mean age of 39.9 (SD 11.4) years, ranging from 19 to 69 years. The average BMI was 23.9 (SD 3.3) kg/m².

The average sleep quality in this population is considered mildly impaired, with a mean PSQI score of 6 out of 21. The SD of 2.9 highlights significant variability.

The most common mattress types were foam (n=41, 35%), spring (n=29, 25%), and latex (n=24, 21%). Around 55% (n=64) of participants reported having a bed partner. Sensor disturbances were reported by 46 (39%) participants due to the PSG device, while no disturbances were reported regarding the WSA nearable.

### Sleep Stages Classification

**Figure 3.**
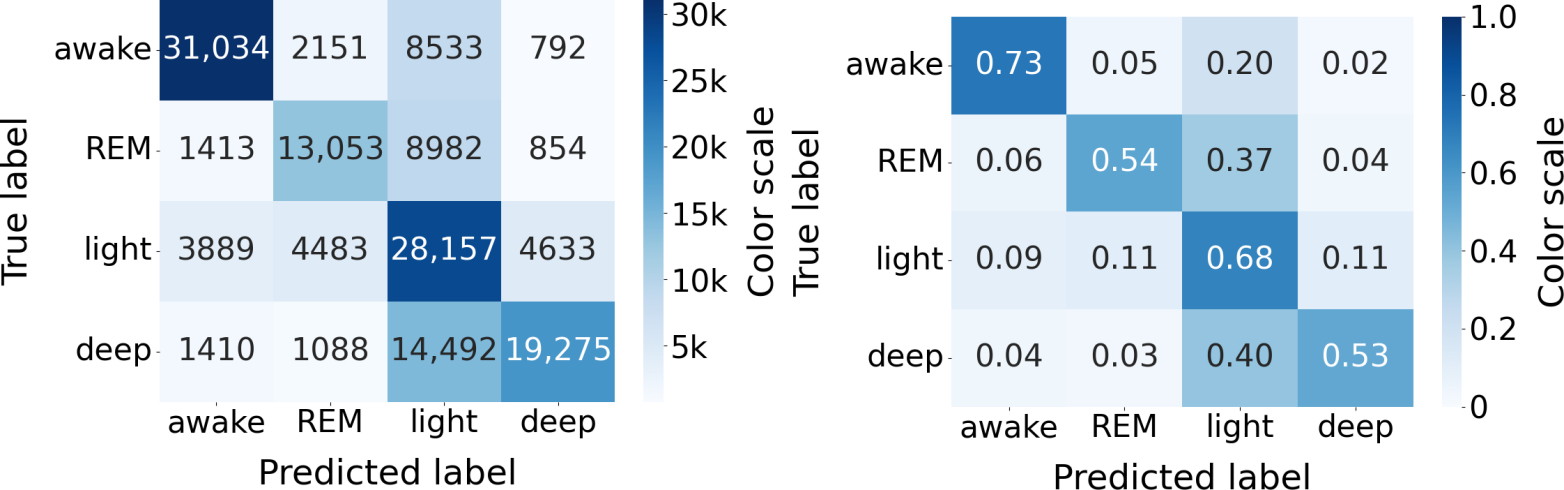
Sleep stage classification confusion matrix (N=117). (Left) Raw confusion matrix displaying total epoch counts for WSA sleep stage detection versus PSG ground truth. (Right) Row-normalized confusion matrix (expressed in percentages) illustrating the classification sensitivity and misclassification rates for each true sleep stage. PSG: polysomnography; REM: rapid eye movement (sleep stage); WSA: Withings Sleep Analyzer.

Figure 3 presents the confusion matrix containing the epochs across all nights, and Table 2 gathers sleep stages classification results on the whole dataset.

**Table 2.** Summary of sleep stage classification results over nights (N=117) (computed as the averages of all nights).

Mean of results per night	Sleep stages								Sleep-wake			
	Kappa	Accuracy	Se^a^ REM^b^	Se light	Se deep	Sp^c^ REM	Sp light	Sp deep	Kappa	Accuracy	Se sleep	Sp sleep
Mean	0.49	0.63	0.53	0.68	0.56	0.93	0.69	0.94	0.65	0.87	0.93	0.73
95% CI	0.47-0.51	0.62-0.65	0.48-0.57	0.65-0.71	0.51-0.60	0.92-0.94	0.67-0.71	0.93-0.95	0.62-0.69	0.86-0.89	0.92-0.94	0.69-0.77
SD	0.12	0.09	0.24	0.16	0.25	0.05	0.11	0.06	0.19	0.07	0.06	0.22

a Se: sensitivity.

b REM: rapid eye movement (sleep stage).

c Sp: specificity.

#### Sensitivity and Specificity

The WSA sensitivity for detecting sleep was 93% (95% CI 92%-94%; N=117), meaning it correctly identifies when a person is asleep 93% of the time. The mean specificity was 73% (95% CI 69%-77%), indicating it accurately detects when a person is awake 73% of the time.

The performance in accurately classifying each individual sleep stage against the other three is more variable, with sensitivities of 53% (95% CI 48%-57%), 68% (95% CI 65%-71%), 56% (95% CI 51%-60%), and 73% (95% CI 69%-77%) for REM, light sleep, deep sleep, and wake stage, respectively. The corresponding specificities were 93% (95% CI 92%-94%), 69% (95% CI 67%-71%), 94% (95% CI 93%-95%), and 93% (95% CI 92%-94%), respectively.

#### Cohen κ and Accuracy

Please note that, contrary to sensitivity and specificity, Cohen κ and accuracy depend on the relative sizes (prevalences) of each class. In this study’s dataset, the WSA mean Cohen κ was 0.65 (95% CI 62%-69%) for distinguishing between sleep and wake stages, and 0.49 (95% CI 47%-51%) for differentiating among the 4 stages (N=117). WSA accuracy to distinguish between sleep and wake was 87% (95% CI 86%-89%). The mean accuracy for the 4 stages was 63% (95% CI 62%-65%).

### Assessment of Sleep Quantity Measures

**Figure 4.**
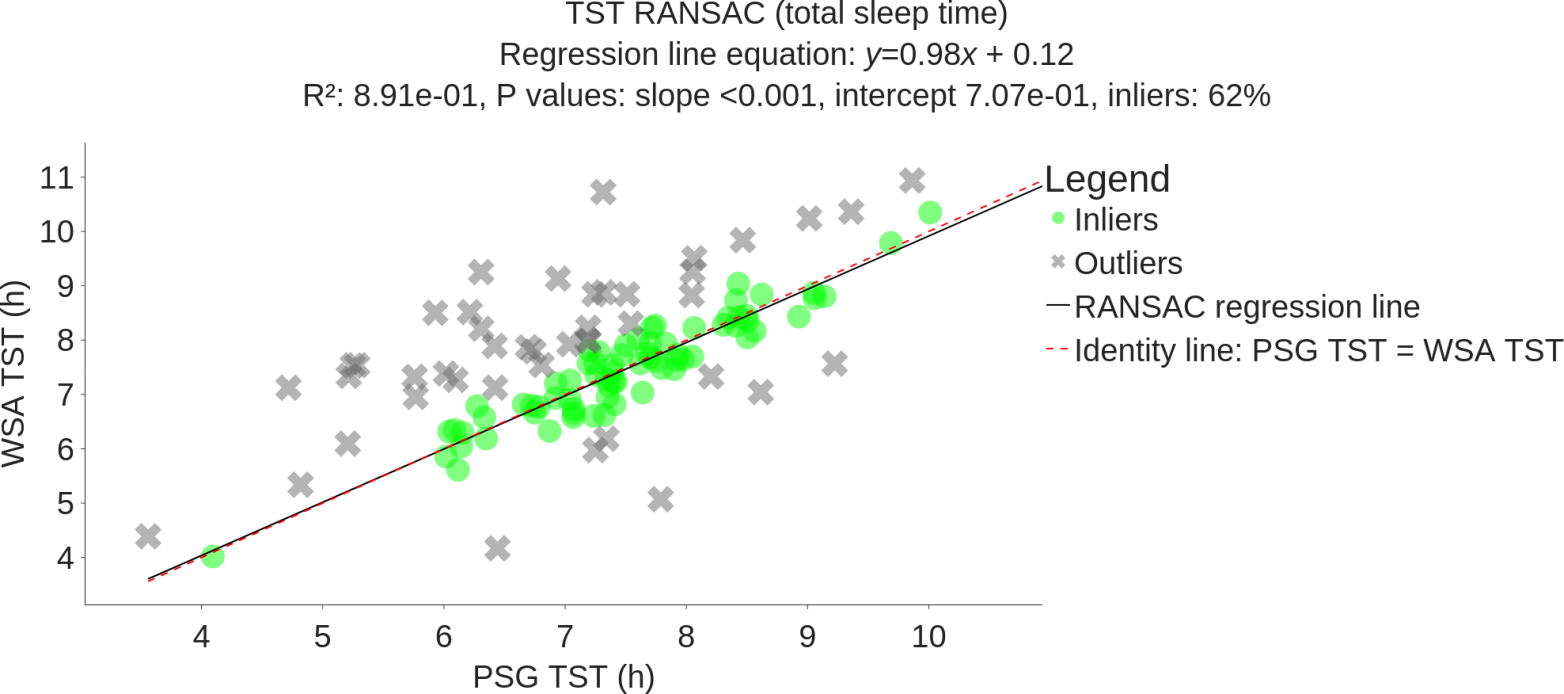
RANSAC regression plot between WSA and PSG total sleep time (black line) compared to the identity line (red dashed line) on 117 participants (*R*^2^=0.89, slope *P* value *P* < .001, inliers: 62%). PSG: polysomnography; RANSAC: random sample consensus algorithm; TST: total sleep time; WSA: Withings Sleep Analyzer.

Figure 4 shows the TST estimated by the WSA versus the PSG. The slope of the linear regression is 0.98 (*P*<.001) and the *R*^2^ value is 0.89, with 38% of outliers, indicating a moderate linear relationship with PSG.

WSA deep sleep was not correlated to time spent in deep sleep (slope estimate of 0.05, *P*=.7, Figure S4 in Multimedia Appendix 1). The WSA estimates of light sleep and REM were moderately correlated with PSG values, with respective slopes of the regression lines of 0.4 (*P*<.001, Figure S5 in Multimedia Appendix 1) and 0.65 (*P*<.001, Figure S6 in Multimedia Appendix 1).

Precision of the WSA estimates is measured with the MAE. The MAE values of total sleep time (equal to the MAE of wake duration because the sum of TST and wake duration is the recording time), REM duration, light sleep duration, and deep sleep duration are 44 min, 35 min, 1 h 33 min, and 1 h 4 min, respectively, with average PSG values of 7 h 15 min (3 h 03 min for awake duration), 1 h 43 min, 2 h 57 min, and 2 h 35 min, respectively. Corresponding 95% CIs are reported in Table 3. The average number of episodes in each sleep stage and the average number of stage changes can be found in Table 4. The WSA tends to underestimate the number of episodes for each sleep stage and is less sensitive to transitions between epochs.

**Table 3.** Summary of Bland-Altman results obtained on sleep-related metrics between WSA^a^ and PSG^b^ estimations (N=117).

Sleep quality measures	PSG value, mean (SD)	MAE^c^ (95% CI)	Bias, WSA – PSG (95% CI)
TST^d^	7 h 15 min (1 h 14 min)	44 min (36 min to 52 min)	20 min (10 min to 31 min)
TIREM^e^	1 h 43 min (45 min)	35 min (30 min to 40 min)	–15 min (–23 min to –8 min)
TILight^f^	2 h 57 min (1 h 12 min)	1 h 32 min (1 h 21 min to 1 h 44 min)	1 h 21 min (1 h 8 min to 1 h 36 min)
TIDeep^g^	2 h 35 min (54 min)	1 h 4 min (55 min to 1 h 13 min)	–46 min (–59 min to –34 min)
TIWake^h^	3 h 3 min (1 h 19 min)	44 min (36 min to 52 min)	–20 min (–31 min to –10 min)
PIREM^i^	17% (8.8%)	5.7% (4.9% to 6.5%)	–2.4% (–3.6% to –1.1%)
PILight^j^	29% (14%)	15% (13% to 17%)	13% (11% to 15%)
PIDeep^k^	25% (11%)	10% (8.8% to 12%)	–7.4% (–9.4% to –5.4%)
PIWake^l^	29% (11%)	7.1% (5.8% to 8.4%)	–3.3% (–5.0% to –1.6%)

a WSA: Withings Sleep Analyzer.

b PSG: polysomnography.

c MAE: mean absolute error.

d TST: total sleep time.

e TIREM: time spent in rapid eye movement sleep stage.

f TILight: time spent in light sleep stage.

g TIDeep: time spent in deep sleep stage.

h TIWake: time spent in wake stage.

i PIREM: proportion of the night in rapid eye movement sleep stage.

j PILight: proportion of the night in light sleep stage.

k PIDeep: proportion of the night in deep sleep stage.

l PIWake: proportion of the night in wake stage.

**Table 4.** Comparison of the number of sleep stage episodes and stage changes between WSA^a^ and PSG^b^ (N=117).

Sleep quality measure	Episodes^c^ of REM^d^	Episodes^c^ of light	Episodes^c^ of deep	Episodes^c^ of wake	Stage changes
WSA, mean (95% CI)	4.1 (3.8-4.5)	8.9 (8.4-9.3)	4.4 (4.0-4.7)	4.4 (4.1-4.8)	8.9 (8.4-9.3)
PSG, mean (95% CI)	5.0 (4.7-5.3)	13.2 (12.7-13.8)	7.1 (6.7-7.6)	4.9 (4.5-5.4)	13.2 (12.7-13.8)

a WSA: Withings Sleep Analyzer.

b PSG: polysomnography.

c An episode is a sequence of consecutive epochs in the same sleep stage.

d REM: rapid eye movement (sleep stage).

Additional scatter plots and regression analyses of these metrics can be found in Section D of Multimedia Appendix 1.

**Figure 5.**
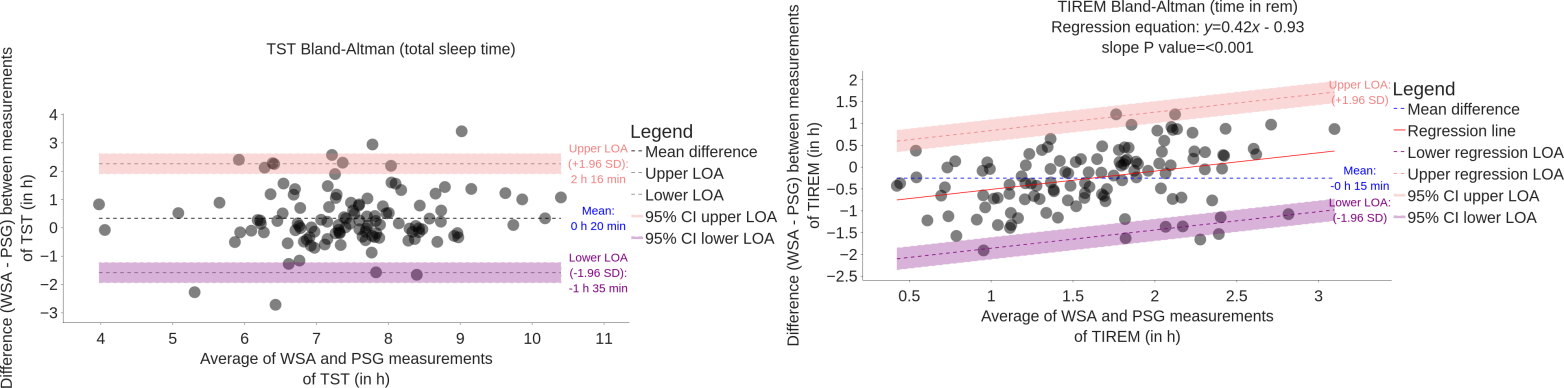
Bland-Altman plot comparing WSA and PSG estimation of total sleep time and time in REM on 117 participants. LOA: limits of agreement; PSG: polysomnography; REM: rapid eye movement (sleep stage); TIREM: time spent in rapid eye movement sleep stage; TST: total sleep time; WSA: Withings Sleep Analyzer.

The Bland-Altman plots are presented in Figures5  6. Biases are summarized in Table 3.

**Figure 6.**
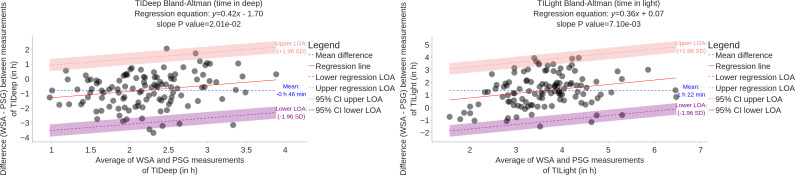
Bland-Altman plot comparing WSA and PSG estimation of time in deep and time in light (N=117). LOA: limits of agreement; PSG: polysomnography; TIDeep: time spent in deep sleep stage; TILight: time spent in light sleep stage; WSA: Withings Sleep Analyzer.

In Figure 5, the Bland-Altman plot for TST does not reveal any proportional bias with the duration of the night. The mean error is 20 minutes. The wide limits of agreement show that there is a substantial amount of variability between PSG and WSA. By contrast, the average error in REM, light, and deep sleep durations depends on the duration of the phase, therefore exhibiting proportional bias. Light sleep is *on average* overestimated for any duration, deep sleep is *on average* underestimated for any duration, and REM is *on average* underestimated for short durations and overestimated for long durations.

The mean error (bias) in light sleep duration is important, with 1 h 21 min. Biases in REM and deep sleep are −15 min and −46 min, respectively. The mean error in TST is therefore 20 min.

### Subgroup Analyses

No significant differences were found in the median or variance of the error across most groups for all the quantitative sleep variables of Table 3. This includes comparisons based on gender, BMI categories, age groups, sleeping arrangements, PSQI scores, self-reported sleep quality, mattress thickness, and night awakenings. However, significant differences in median total sleep time and wakefulness proportion were observed within the PSQI sleep duration component subgroup. Median MAE of TST is respectively 21 min and 1 h 11 min for good and bad sleep duration subgroups, and median MAE of wakefulness proportion is respectively 3% and 12% for good and bad sleep duration subgroups (N=117). This finding suggests that distinguishing sleep from wake is a harder task for the WSA when the participant has subjective short sleep duration (<6 h), highlighting an area for further investigation. For a comprehensive overview of the statistical tests and detailed results, please refer to Multimedia Appendix 2.

**Figure 7.**
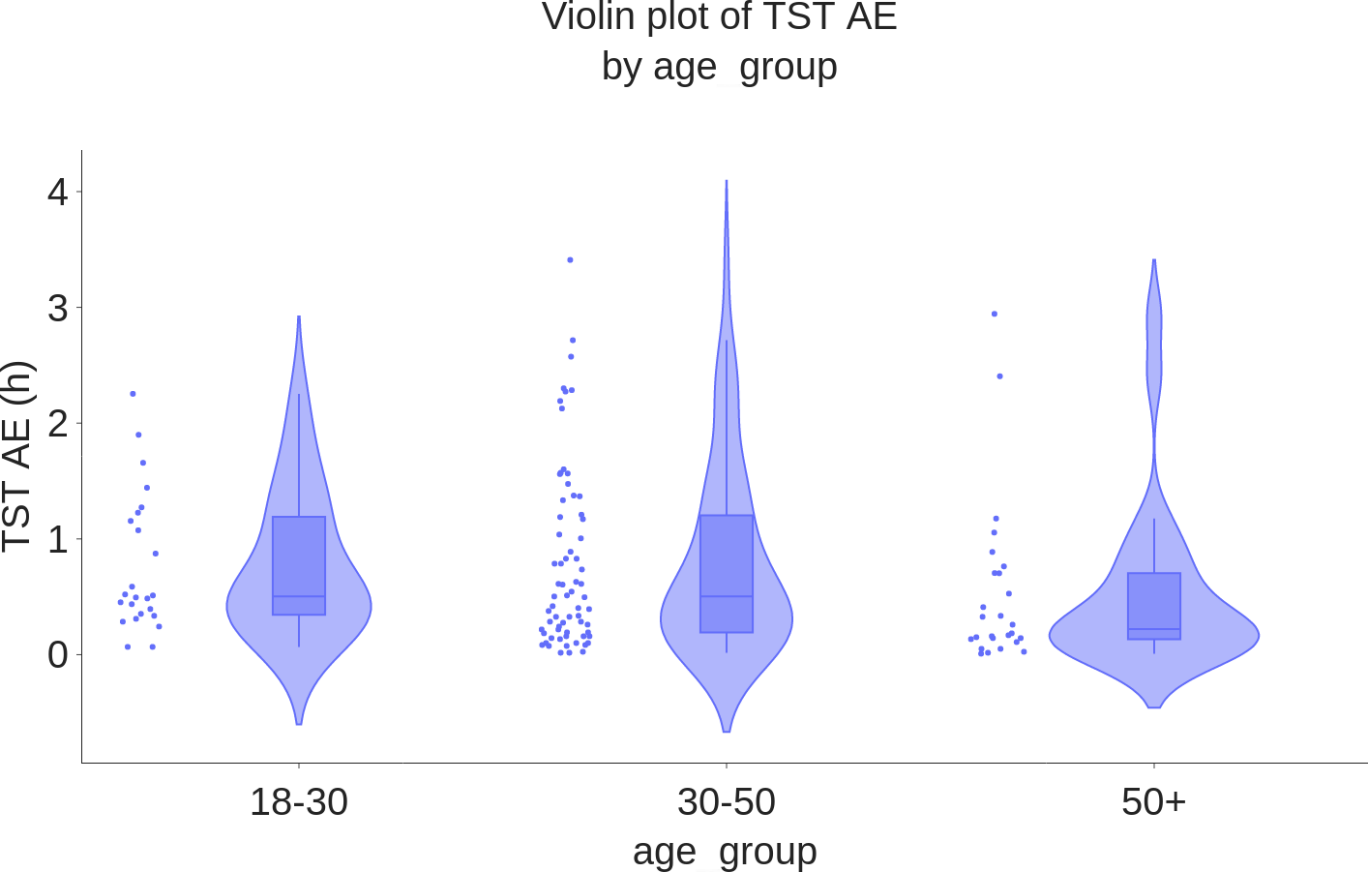
Boxplot of TST mean AE (hours) by age group (N=117). AE: absolute error; TST: total sleep time.

**Figure 8.**
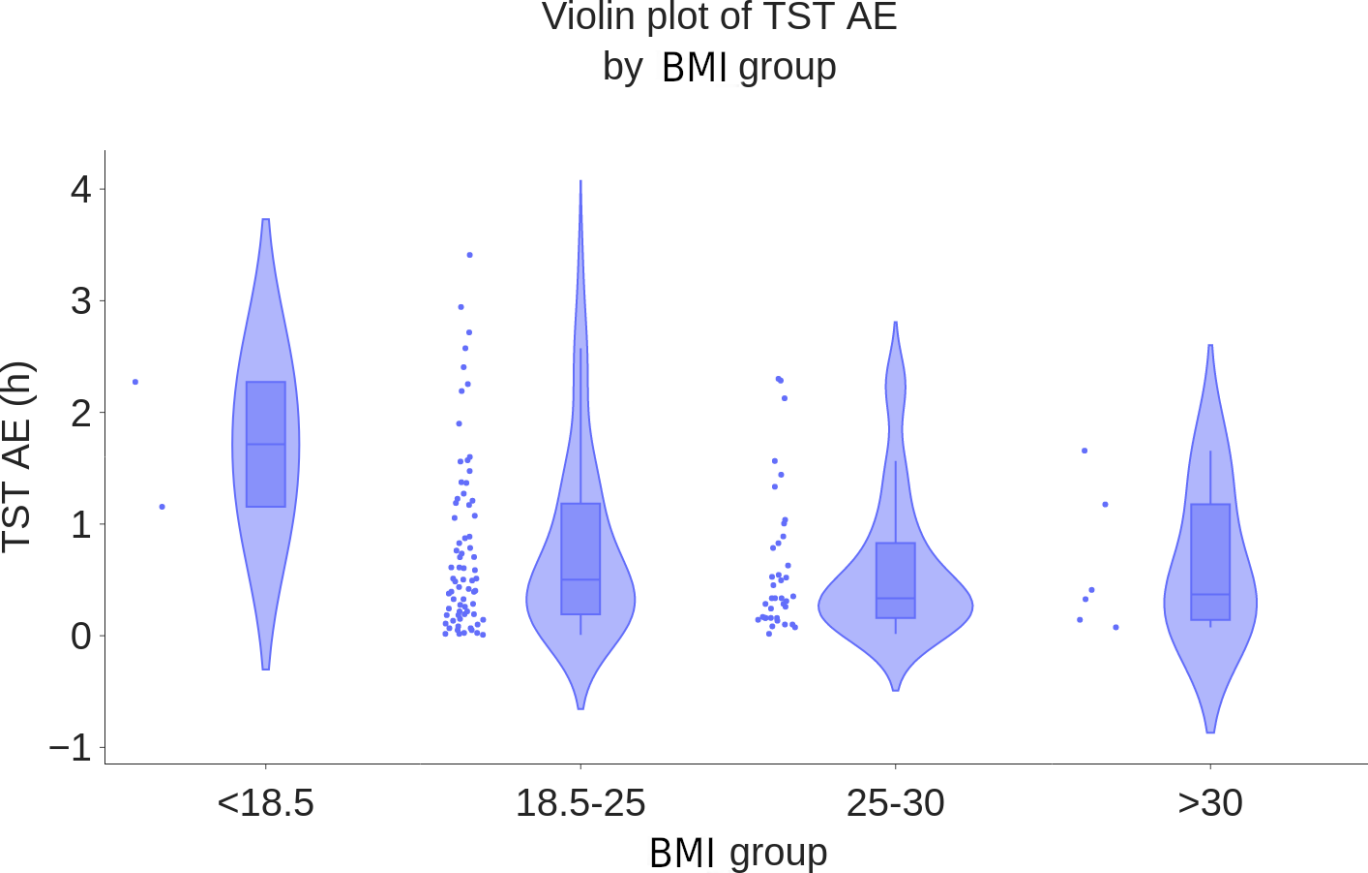
Boxplot of total sleep time mean AE (hours) by BMI group (N=117). AE: absolute error; TST: total sleep time.

**Figure 9.**
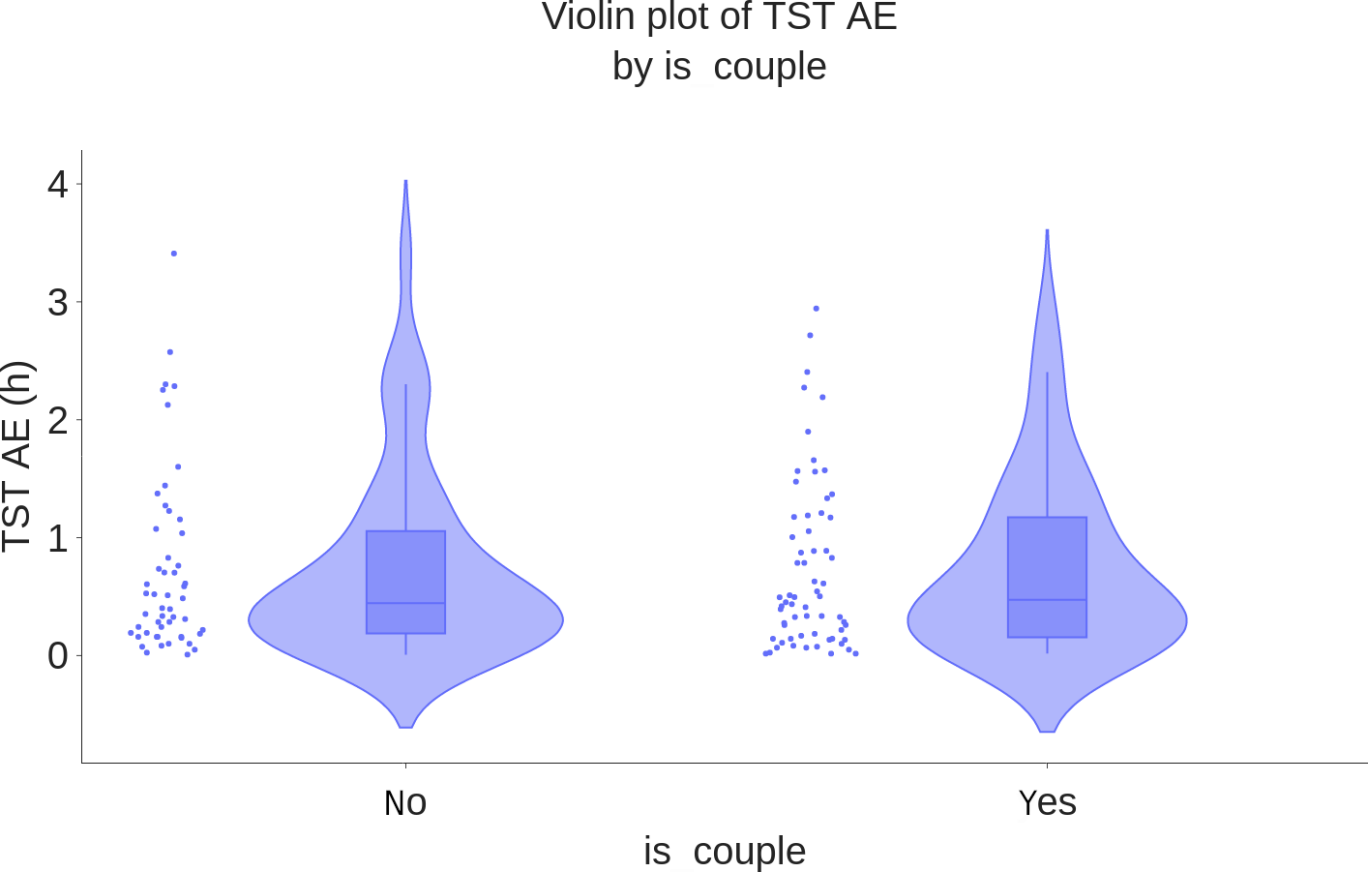
Boxplot of total sleep time mean AE (hours) by *is couple* group (N=117). No: participants who slept alone the night of the recording; yes: participants who had a sleep partner the night of the recording. AE: absolute error; TST: total sleep time.

As an illustration, Figures 7-9 show the boxplots of TST MAE of WSA compared to PSG, stratified by age groups, BMI ranges, and whether the participant was alone in bed. They suggest that the WSA performance did not depend on these factors. Boxplots for TST MAE for the other subgroups can be found in Section D of Multimedia Appendix 1.

### Agreement Between Reviewers

Each 30-second epoch of the PSG data was independently annotated by two reviewers. The mean κ value for sleep stage determination between reviewers before reaching consensus is 0.71 (SD 0.11), closely aligning with literature findings (κ=0.76 in [53]), while it is 0.81 (SD 0.17) for sleep-wake classification.

**Figure 10.**
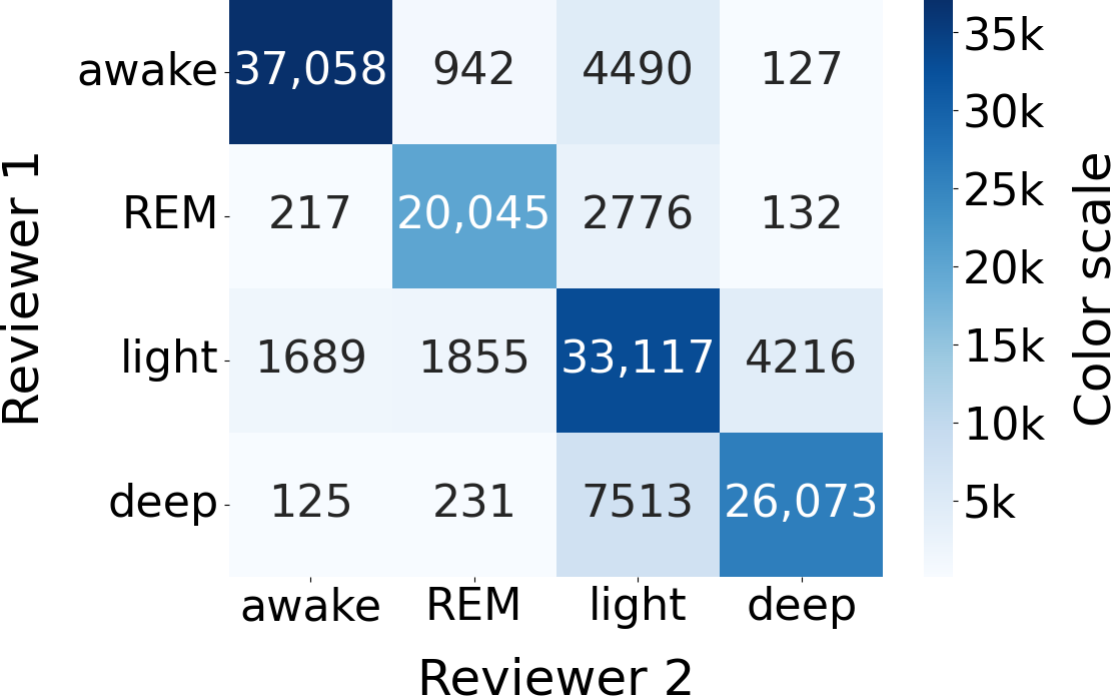
Sleep stage classification confusion matrix between the first two reviewers’ annotations (N=117). REM: rapid eye movement (sleep stage).

In the analysis of reviewer agreement on sleep stage classifications, the primary disagreements occur between light and deep stages, as well as between light sleep and awake/REM stages (Figure 10). This indicates that even expert reviewers, considered the gold standard, face challenges in accurately distinguishing these stages due to their inherent similarities and subtle physiological differences. This inherent difficulty in distinguishing light, deep, awake, and REM sleep stages by human experts is reflected in WSA, which was trained on PSG data labeled using a similar protocol. It is likely that the imprecision of the gold standard is passed on to the device.

#### Discrepancies in Performance: Epochs With Reviewers’ Agreement vs Disagreement

Given the discrepancies encountered in PSG annotation among reviewers, it is pertinent to investigate whether the WSA performed better on epochs where reviewers reached consensus compared to those where disagreements were present. To perform this analysis, the dataset was divided into two groups of epochs: those where reviewers reached agreement and those where disagreements occurred. Separate performance analyses were then conducted on these two complementary groups.

**Figure 11.**
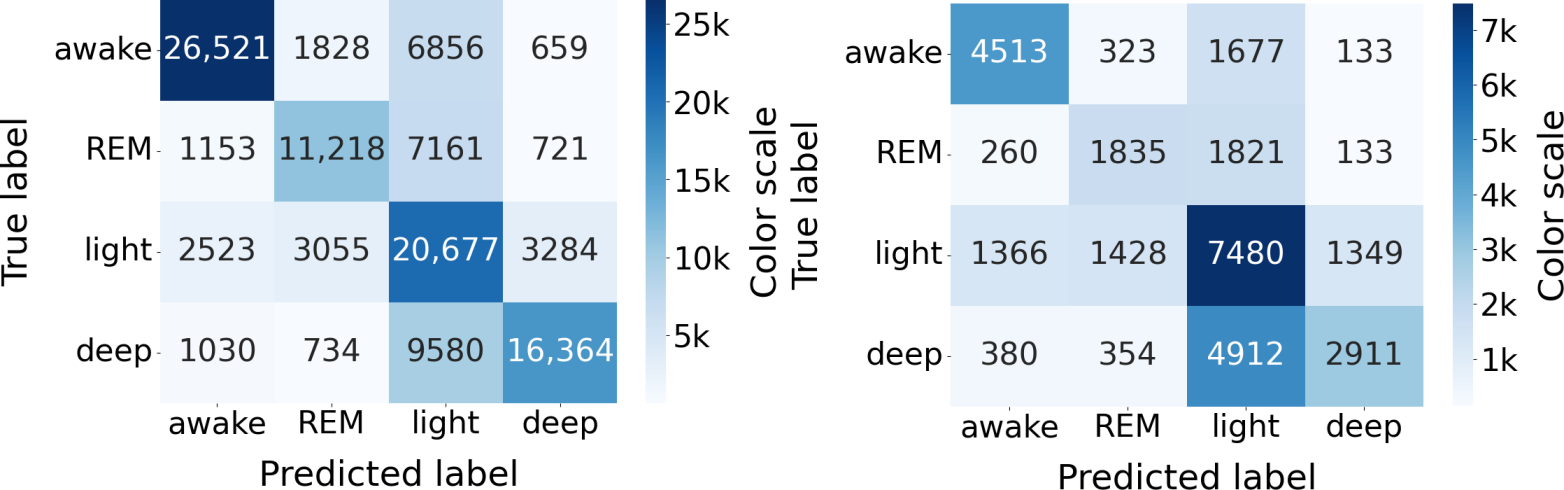
Sleep stage classification confusion matrices (N=117) on sets with agreement (left) and disagreement (right); WSA sleep stage detection vs PSG ground truth. PSG: polysomnography; REM: rapid eye movement (sleep stage); WSA: Withings Sleep Analyzer.

Results are summarized in Table 5. In the “agreement” column (resp. “disagreement” column), only epochs where both reviewers agreed (resp. disagreed) were included, while epochs with disagreement (resp. agreement) were excluded. Figure 11 shows the confusion matrices obtained for both sets.

**Table 5.** Summary of performance in reviewers’ agreement and disagreement subgroups (N=117).

WSA^a^ vs PSG^b^	Agreement	Disagreement	*z* score
Sleep stages			
Epochs, n (%)	113,364 (79)	30,875 (21)	—^c^
Kappa, mean (95% CI)	0.52 (0.50‐0.55)	0.27 (0.24‐0.30)	78.06
Accuracy, mean% (95% CI)	0.66 (0.64‐0.68)	0.53 (0.50‐0.55)	42.00
Se^d^ REM^e^, mean (95% CI)	0.55 (0.50‐0.59)	0.44 (0.39‐0.49)	34.32
Se light, mean (95% CI)	0.7 (0.67‐0.72)	0.64 (0.60‐0.67)	20.16
Se deep, mean (95% CI)	0.56 (0.51‐0.60)	0.37 (0.32‐0.43)	68.80
Se wake, mean (95% CI)	0.72 (0.67‐0.76)	0.51 (0.44‐0.58)	69.85
Sp^f^ REM, mean (95% CI)	0.94 (0.93‐0.95)	0.92 (0.90‐0.93)	12.70
Sp light, mean (95% CI)	0.72 (0.69‐0.74)	0.52 (0.48‐0.56)	66.63
Sp deep, mean (95% CI)	0.95 (0.94‐0.96)	0.92 (0.90‐0.94)	20.25
Sp wake, mean (95% CI)	0.94 (0.93‐0.95)	0.92 (0.90‐0.93)	12.70
Sleep-wake			
Epochs, n (%)	133,447 (93)	10,792 (7)	—
Kappa, mean (95% CI)	0.64 (0.59‐0.68)	0.33 (0.26‐0.38)	63.72
Accuracy, mean% (95% CI)	0.88 (0.86‐0.89)	0.76 (0.71‐0.78)	35.77
Se wake, mean (95% CI)	0.74 (0.67‐0.76)	0.52 (0.46‐0.59)	49.15
Se sleep, mean (95% CI)	0.94 (0.93‐0.95)	0.86 (0.83‐0.89)	32.20

a WSA: Withings Sleep Analyzer.

b PSG: polysomnography.

c Not applicable.

d Se: sensitivity.

e REM: rapid eye movement (sleep stage).

f Sp: specificity.

We used a *z* test for proportions to test for differences between sensitivity, specificity, accuracy, and kappa. All the results are statistically significant, with WSA performing better on epochs where the two reviewers agreed. The progression is particularly significant for the sensitivity in deep sleep, reaching 0.56, compared to 0.37 when the reviewers disagreed, as in the latter case, WSA predominantly classified deep sleep stages as light sleep. The specificity for light sleep was also starkly improved from 0.52 to 0.72. As a result, the Cohen κ is almost double when reviewers agreed (0.64 vs 0.33 for S/W classification, and 0.52 vs 0.27 for 4-stage classification).

### Sleep Quality With vs Without PSG

Since this analysis does not involve the WSA or the PSG data, but only the participants’ answers to the questionnaires, it was conducted on the 186 participants for whom reported sleep quality data were available. Results are given in Table 6.

**Table 6.** Comparison of reported sleep quality and sensor disturbance with and without PSG^a^ (n=186). In both cases, the participant used a WSA^b^ and two ScanWatch devices.

Qualitative sleep quality measures	First night with PSG	Second night without PSG
Sleep quality, n (%)		
Very poor	10 (5.4)	3 (1.6)
Poor	65 (34.9)	32 (17.2)
Quite good	75 (40.3)	71 (38.2)
Good	32 (17.2)	58 (31.2)
Very good	4 (2.2)	22 (11.8)
Sleep quality compared to usual sleep quality, n (%)		
Much worse	28 (15.1)	6 (3.2)
Worse	69 (37.1)	32 (17.2)
Same	77 (41.4)	111 (59.7)
Better	10 (5.4)	31 (16.7)
Much better	2 (1.1)	6 (3.2)
Sensor disturbance		
Device that caused sleep disturbances	PSG	WSA
Yes, n (%)	77 (41.4)	0 (0)
No, n (%)	109 (58.6)	0 (0)

a PSG: polysomnography.

b WSA: Withings Sleep Analyzer.

Despite the removal of the nasal cannula and the blood oximeter from our home PSG test, the sleep test was associated with significant discomfort in 41% (77/186) of the participants during the night of the PSG home test, compared to 0% (0/186) in the subsequent night with the WSA only. Fifty-two percent (97/186) (resp. 20%, 38/186) of the participants reported a degraded sleep quality on the night of the PSG (resp. without the PSG).

**Figure 12.**
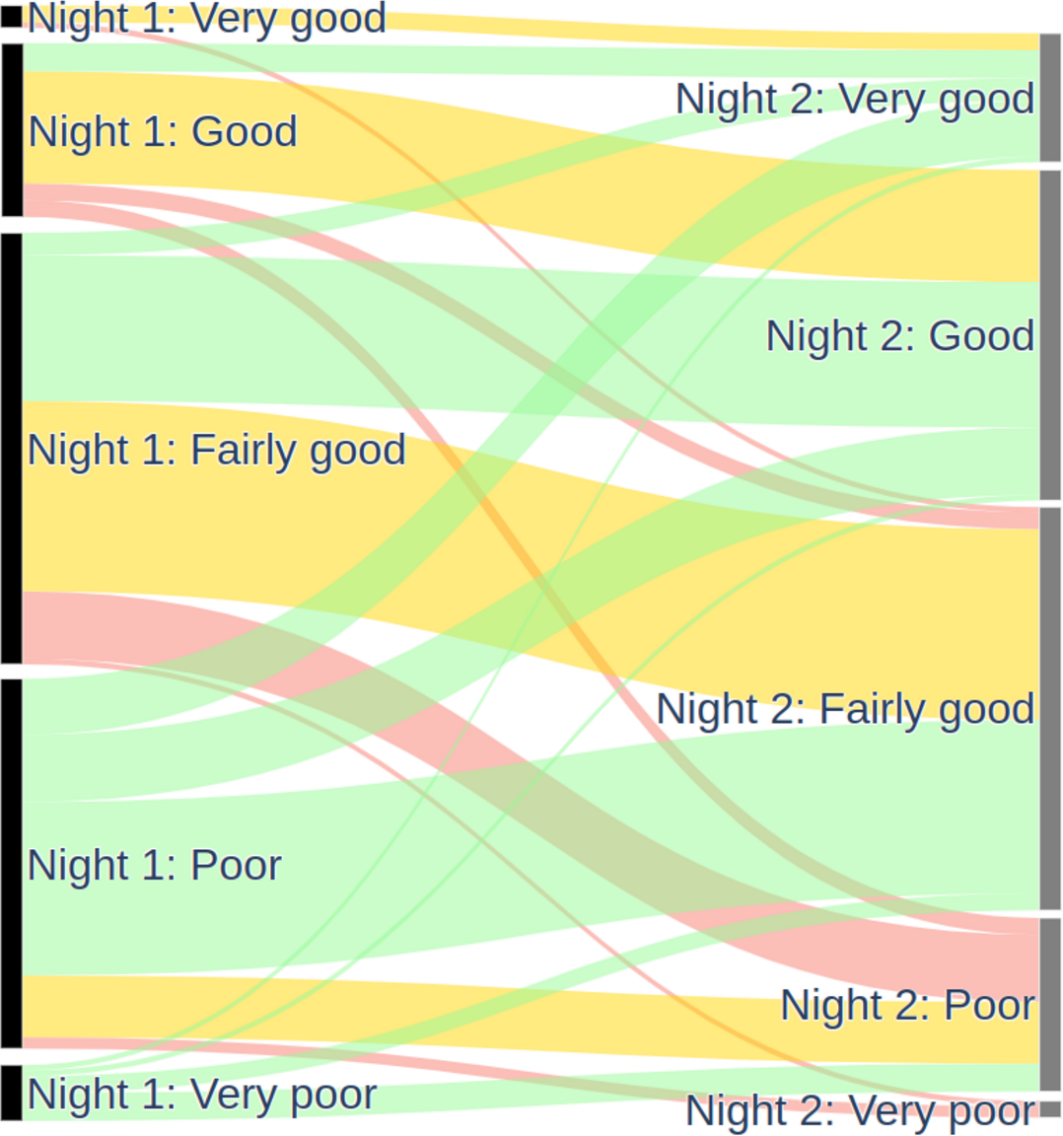
Comparison of reported sleep quality with and without PSG (N=186). Green: improved; yellow: identical; red: deteriorated; black: first night with PSG; grey: second night without PSG. In both cases, the participant used a WSA and two ScanWatch devices. PSG: polysomnography; WSA: Withings Sleep Analyzer.

Figure 12 illustrates the changes in sleep quality among participants over 2 consecutive nights. Participants generally experienced better sleep without the PSG sensors. Notable improvements were observed in 52% (97/186) of cases, with transitions from fairly good to good (26/186, 14%), and from poor to fairly good (31/186, 17%), good (12/186, 6%), or very good (10/186, 5%). Additionally, some participants reported significant improvements from very poor to very good sleep, and others from poor to fairly good. These improvements may be influenced by several factors, including the removal of PSG sensors, which can be intrusive and uncomfortable, potentially disrupting sleep. Furthermore, the awareness of being recorded might alter participants’ sleep behavior, either positively or negatively. The use of unfamiliar devices could also play a role, as participants may initially experience discomfort or anxiety, which diminishes over time. External conditions such as noise and room temperature, which were not controlled in this study, could also significantly impact sleep quality. Therefore, while the data suggest improvements, it is crucial to consider these potential confounding factors when interpreting the results.

Table 6 highlights that participants slept as well with WSA as they typically do. On the second night, 59.7% (111/186) of participants reported the same sleep quality, 16.7% (31/186) reported better sleep, and 3.2% (6/186) reported much better sleep compared to their usual experience. This suggests that Withings sleep monitoring devices do not negatively impact sleep quality and may even enhance it for some users.

## Discussion

### Main Findings

The evaluation of the WSA for sleep stage detection in a cohort of 117 participants in a home setting revealed several key insights. The device demonstrated a high sensitivity of 93% for detecting sleep, underscoring its strong capability to accurately identify sleep periods. However, the specificity was moderate at 73%. The duration of the recording before actual sleep onset and after wake-up significantly affects observed performance of wake detection, with shorter periods inflating specificity, accuracy, and Cohen κ. This occurs due to a shortening of quiet wake periods hard to distinguish from sleep (reading or screen time in bed, for instance), leading to fewer false positives while true negatives remain relatively constant. Conversely, overly long (eg, 24-hour) recordings, dominated by easily identifiable diurnal wakefulness, also tend to inflate specificity. In both cases, the performance of the evaluated device is overestimated. The most clinically relevant and challenging scenario for performance evaluation involves analyzing data encompassing the entire in-bed period, including calm wakefulness before sleep onset and after awakening. While AASM guidelines suggest using lights-off and lights-on as proxies for night boundaries, this approach truncates crucial pre– and post–sleep wake data, therefore introducing a bias in TST and classification performance endpoints. To mitigate this, we used all available PSG annotations, irrespective of lights-off and lights-on times, to provide a more comprehensive and unbiased assessment of performance across the full in-bed duration.

The classification of sleep stages (into wake, REM, light, and deep phases) is more challenging. The confusion matrix (Figure 3) shows that 40% of deep sleep phases, 37% of REM sleep, and 20% of wake phases were misclassified as light sleep by the WSA. As a result, the corresponding sensitivities were rather low (0.56, 0.53, and 0.73, respectively). Thirty-two percent of light sleep stages were misclassified, in similar proportions, into REM, deep sleep, and wake. In consequence, (1) the REM and deep sleep total durations were underestimated (by approximately 30% and 15%), while light sleep duration was overestimated by almost 48% (biases in Table 3), and (2) the specificities for wake, REM, and deep sleep are very high (0.93, 0.94, and 0.94, respectively), while the specificity for light sleep is moderate (0.69).

We showed that these misclassifications of WSA mirror the disagreements between PSG reviewers, who mainly disagree on light versus all the other sleep stages (Figure 11). WSA classification was indeed significantly better on epochs where reviewers agreed (79% of the epochs) than on epochs where they disagreed. WSA’s sleep stage classification uses an algorithm trained by supervised learning on PSG data annotated by technician experts, so it is expected that WSA performance is limited by the interreader variability.

Consistent with existing validation studies [33,44], we also report accuracy and Cohen κ. We caution that these performance metrics are strongly dependent on the prevalence of the classes (here, wake, sleep, REM, deep, and light), which creates difficulties in comparing results between studies. In particular, for sleep studies, two factors modify the prevalence of the classes significantly. First, there is no standardized definition of the beginning of the night. Second, the choice of the reference device has some importance, causing more or less sleep disturbances.

With these caveats in mind, the WSA achieved an overall accuracy of 87% for sleep-wake distinction, which is promising for a contactless home monitoring device. Despite this, the accuracy for classifying specific sleep stages was moderate, with REM sleep at 53%, light sleep at 68%, and deep sleep at 56%. The overall accuracy for sleep stage classification was 63%, with a Cohen κ of 0.49.

Notably, the WSA’s performance was consistent across various demographic subgroups, including different age and BMI categories. Importantly for a device under the mattress, there was no difference between individuals who slept alone and those with a bed partner, nor between different types or thicknesses of mattresses. Other groups analyzed included sex, PSQI scores, self-reported sleep quality, and whether individuals woke up during the night, all of which also showed no significant differences in MAE, except for wake proportion by subjective sleep duration group. This consistency suggests the device may be useful for a wide range of participants.

### Comparison With Previous Research

#### Differences in Studies’ Protocols

To contextualize our findings, we used published validation studies of other consumer sleep trackers. We advise interpreting these comparisons with caution, as study protocols vary significantly (eg, in-lab vs at-home settings, population characteristics, and data handling procedures). We compare our results on WSA with influential studies that have contributed to the understanding of consumer sleep-tracking technology. Studies [29], [37], and [33], respectively, evaluated 7, 5, and 6 consumer devices against PSG, mainly wearable devices, revealing significant variability in their accuracy for sleep stage detection. Studies [33,54-60] compared a total of 8 contact-free devices and 1 wearable against PSG. These studies provide a valuable context for evaluating our device’s performance, illustrating both the advancements and challenges in the field of consumer sleep technology. All the data used for this comparison can be found in Multimedia Appendix 3.

We compared the performance metrics reported by other studies on their respective datasets to the performance we obtained on our dataset, without reproducing their results or running their device algorithms on our data. When a result was not explicitly provided in the study report, we calculated it from the confusion matrix if it was available.

Consistent with recent validations [39], our study population encompasses a diverse range of cofactors of sleep quality, presenting a wide range of age and BMI, as was the case in only 2 of the 11 studies used for the comparison [55,59]. Four studies [37,38,44,58] included only young adults, and one study focused on older adults [33].

The conditions of our study are not controlled since participants stayed at home, as in only one other study [54] but on a much smaller scale (n=20). Our dataset is therefore richer regarding important environmental cofactors of sleep quality, including sleeping arrangements (alone or with a partner), mattress types, ambient temperature, lighting and sound, prebedtime activities (eg, no restriction on caffeine or alcohol consumption, on screen time, no forced sleep regularity), and activities during time in bed (eg, no forced turning off of the lights).

In addition, some studies use strict exclusion criteria also after data collection [38] to ensure data quality and reliability, such as excluding individual nights with a total recording time of less than 7 hours and 50 minutes [37].

Thus, our real-world home setting captures the natural variability in sleep patterns and environmental factors. While this enhances the generalizability of our results, reflecting typical user experiences, it also presents a less favorable scenario for the device, posing additional challenges for accurate sleep stage detection and analysis (for instance, activities in bed like reading or screen time can be confounded by the device as sleep). Consequently, our findings provide a more realistic evaluation of the device’s performance in everyday conditions.

#### Sleep-Wake Classification

**Figure 13.**
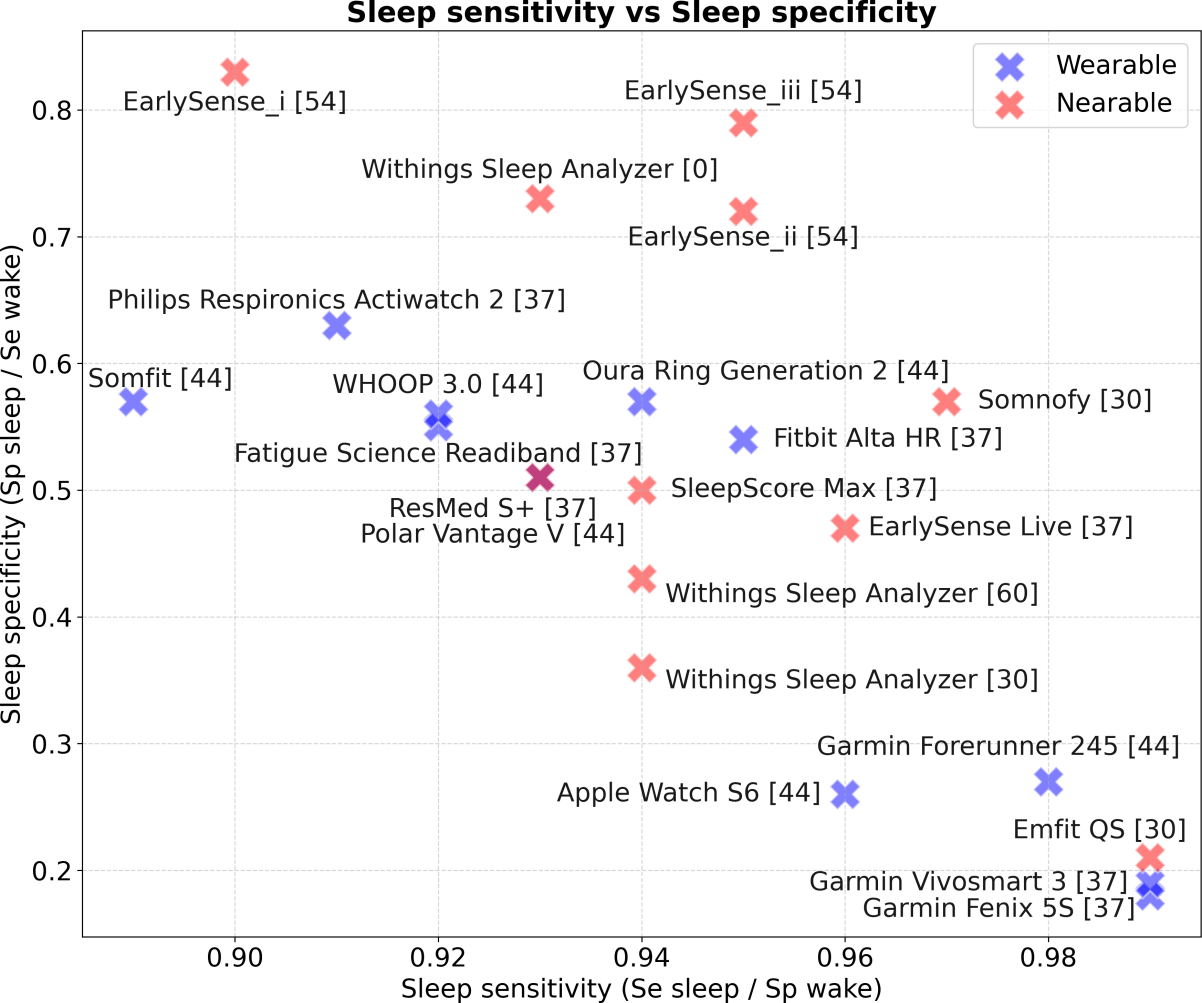
Overview of sleep-wake reported sensitivities from varying validation studies. Indication [0] denotes this study. Note: Direct comparison is limited by differences in study protocols and populations [33,37,44,54,60]. The exact data points corresponding to the figure are fully detailed in Multimedia Appendix 3.

The choice of a sleep monitoring device requires being informed about the trade-off between sleep and wake identification. This is illustrated in Figure 13. Devices with high sleep sensitivity (≥0.98) exhibit low specificity (<0.30), as observed with the Garmin Forerunner 245, Emfit QS [33], Garmin Vivosmart 3, and Garmin Fenix S5. Conversely, devices achieving higher specificity (≥0.70), such as the EarlySense [54] and WSA, tend to show slightly lower sensitivity (0.90‐0.95).

As explained above, the proportion of wake stages in the dataset before and after sleep onset can significantly impact the sleep/wake endpoints. These studies vary in the proportions of wake epochs, which refer to the total time spent awake divided by the total recording duration of the entire dataset. Specifically, these proportions ranged from 8% to 14% in [37], 15% in [54], ranged from 20% to 33% in [44], and from 34% to 42% in [33], compared to 30% in our dataset. This information was unfortunately not available for most of the studies evaluating nearable devices [55-60]. Lower proportions of wake data can inflate overall accuracies and kappas if sleep epoch classification dominates, as noted in [61]. In datasets with a low proportion of wake epochs, overall accuracy and kappa can be misleadingly high due to the dominance of sleep epoch classification, which is better classified than wake epochs as indicated in the previous paragraph.

#### Sleep Stages Classification

**Figure 14.**
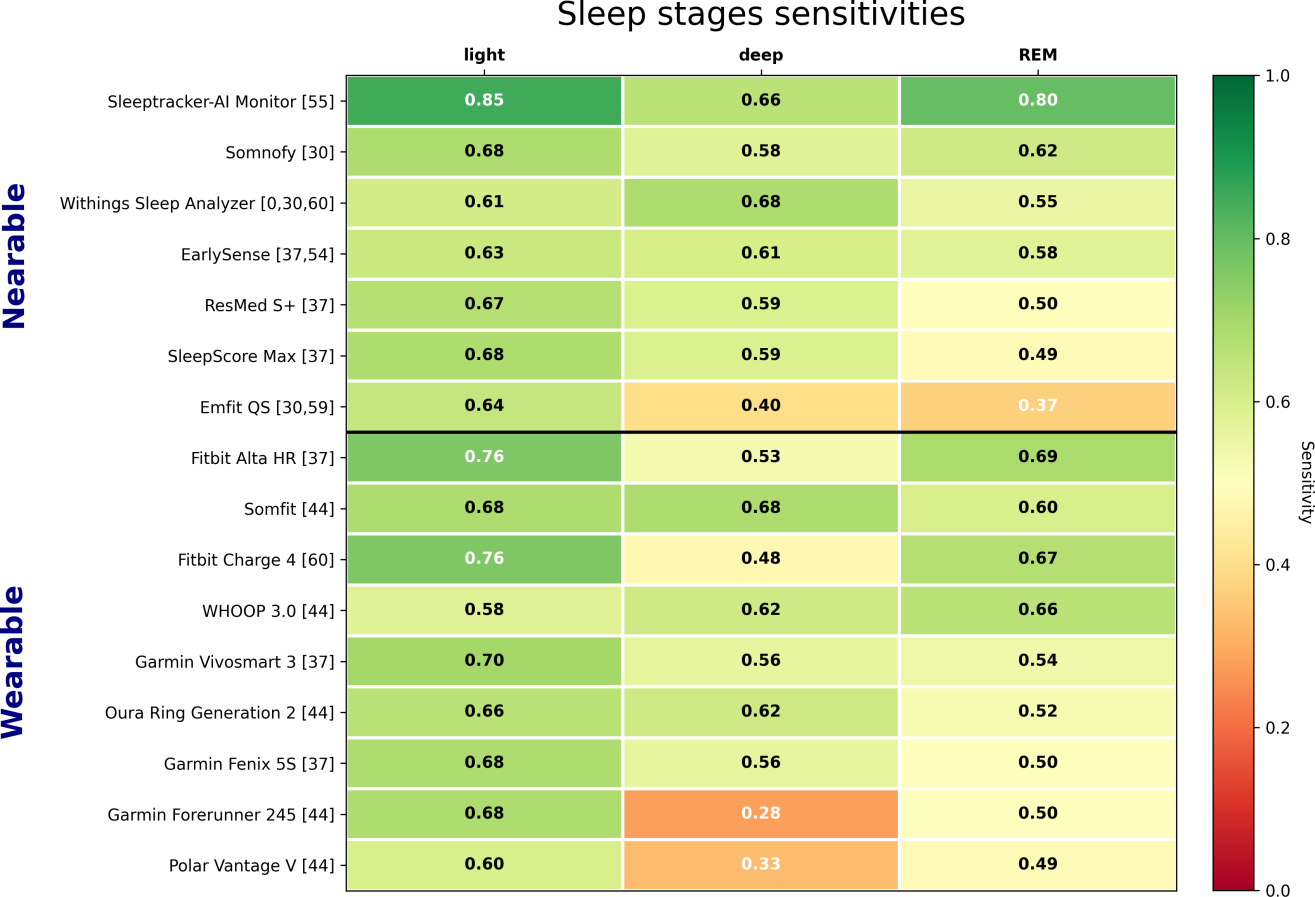
Overview of sleep stage sensitivities from varying validation studies. Indication [0] denotes this study. Note: Direct comparison is limited by differences in study protocols and populations [33,37,44,54,55,59,60].

The trade-off observed in sleep-wake classification is even more pronounced in sleep stage analysis, where no single device consistently outperforms the others across all stages (Figure 14). The range of sensitivities is particularly large for REM, from 0.37 to 0.80, and quite large for deep sleep (0.28 to 0.68), even after excluding wearables (0.40 to 0.68). The sensitivities for light sleep detection are more homogeneous, with 12 sensitivities between 0.6 and 0.7. The WSA is in the top tier of devices for deep sleep classification, but midrange for light sleep and REM.

The confusion matrices show that, for all devices, the highest proportion of misclassification for each stage is wrong light sleep detection, which can be explained by the inherent difficulty in distinguishing light from the other sleep stages by human experts, as shown in the Results section.

#### Device Comparison Conclusion

This analysis highlights the necessity of considering a comprehensive set of metrics to accurately evaluate the capabilities of each device. Relying on isolated overall metrics such as accuracy or kappa is insufficient to provide a fair assessment [62,63]. Every device exhibits relative strengths and weaknesses, and no single device clearly outperforms the others across all tasks. The WSA specifically demonstrates robust performance in sleep-wake distinction, while the precise identification of individual sleep stages remains a more challenging task.

### Methodological Aspects and Strengths of the Study

The rapid adoption of new sleep technologies in consumer and clinical settings demands rigorous performance evaluation [64]. To improve the reliability and comparability of sleep research, we describe our methodology in detail and with transparency, an approach that is supported by recent guidelines advocating for uniform analysis pipelines [41,42,44]. Our study was designed to resolve methodological issues frequently found in the literature.

The study’s strength lies first in its diverse sample of over 100 participants, who are for the most part healthy and with diverse demographic characteristics. Additionally, the inclusion of relevant factors that may negatively impact the device’s performance, like the presence of a bed partner and variations in mattress type and thickness, further enriches the dataset, allowing for a comprehensive evaluation of the device’s robustness across different real-life conditions in a healthy population. This allowed us to conduct meaningful subgroup analyses.

A second strength of the study is the method of PSG analysis. Unlike [44,55-58], which opted for a single reviewer, or two reviewers without consensus as in [37], our study is aligned with [33,38] by implementing a double review process with adjudication by a third reviewer in case of disagreement. This consensus methodology is preferable since it enhances the reliability of the sleep stage classification. In particular, we have shown that reviewers often err by misclassifying light sleep stages, a finding consistent with previous research reporting that the interrater reliabilities for light and deep stages were moderate [61]. A reliable assessment of devices’ performance in sleep stage classification therefore requires a strong methodology to establish the ground truth.

Third, with a view to limit the participants’ discomfort, we chose to perform PSG at home with a limited number of sensors, without the nasal cannula and oximeter. This setup reduces the sleep disturbances typically associated with lab-based PSG, allowing for a more natural assessment of sleep patterns. This limited set of sensors is sufficient for the evaluation of TST and sleep stage classification, with the trade-off that sleep onset, time in bed, and wakefulness after sleep onset cannot be assessed. Yet, significant sleep disturbances were reported by the participant during the PSG test, underscoring the challenges of completely undisturbed monitoring even in home environments. Despite this, the study offers a realistic evaluation of the WSA’s capabilities under typical sleeping conditions.

Fourth, we aimed to give a comprehensive report of the device’s performance. Collecting epoch-by-epoch data enables a granular examination of sleep patterns, the consistency of measurement errors over time, and thus enables an accurate assessment of the reliability and utility of sleep monitoring devices in real-world settings. We emphasized the need for such comprehensive reporting and the elements of the protocol that may bias the performance estimates. Thus, transparency requires a presentation of the results including all the performance endpoints, each presenting a different facet of the device’s performance [41-43].

In conclusion, given these methodological strengths, our study provides valuable insights into the WSA sleep stage algorithm’s performance in real-world settings.

### Limitations and Future Work

Despite our efforts for a solid methodology, the study faced several challenges.

#### Challenges in Defining Bedtime and Wake Time in Sleep Studies

A first challenge is created by the lack of a standardized definition of the start time and end time of a sleep study. As per AASM guidelines [50], bedtime and wake time are defined as the “lights-off” and “lights-on,” respectively. This is not adapted to studies in real-world settings, where participants often go to bed and engage in activities before attempting to fall asleep. In everyday life, individuals may not turn off the lights immediately upon going to bed, nor do they always intend to sleep right away. Similarly, in the morning, people may not turn on the lights immediately upon awakening, and their actual wake time may not coincide with the “lights-on” reference. This discrepancy is compounded by the lack of a position sensor, which makes it difficult to accurately determine when a person intends to sleep. In other studies [37], start and end times of sleep are determined by the participants themselves, as suggested in standard sleep diaries [65], but the precision of this method is poor.

This lack of standardization is detrimental to the assessment of nearable and wearable devices intended to be used in home settings. Errors in sleep estimation, where wakefulness is mistakenly classified as sleep, are more frequent at the beginning and end of the night. The reason is that many devices, including the WSA, rely heavily on movement detection to distinguish wake from sleep. Thus, wake periods when the participant is immobile and calm in bed, such as when reading or scrolling with a low heart rate, are frequently mistaken as sleep by the device.

Additionally, the practice of activating the PSG immediately before sleep and deactivating it early prevents the gathering of comprehensive data on a participant’s sleep habits. This includes behaviors such as spending extended periods in bed without the intention to sleep. To address this limitation, future studies could extend the duration of PSG recordings to capture activities that occur between the time a person gets into bed and falls asleep, as well as between waking and getting out of bed. This approach would provide a more complete picture of sleep behaviors and enable a thorough evaluation of sleep stage classification algorithms in real-world settings.

#### Challenges in Data Collection From a Nearable

A second challenge in studies with connected devices is data loss. Our study is no exception since nearly 19% of the data were lost, primarily due to the wireless transfer by WSA of a large amount of raw sensor data (250 samples per second). Note that this high rate is not representative of the intended use of the device, which, in its commercial version, only transfers processed data at a low frequency. To mitigate the risk of data loss, Wi-Fi routers paired with the WSA were installed at the participant’s home. The routers experienced occasional connectivity problems. Data loss was further exacerbated by issues such as accidental unplugging of the device and improper reconnection without calibration. These technical challenges underscore the difficulties of gathering high-quality, high-frequency data in uncontrolled settings.

Another factor impacted the accuracy of sleep stage classification; despite receiving instructions from a technician, participants with large beds did not always sleep above the sensor but on the other side of the bed. In such cases, the WSA is not operated optimally, which may have restricted its performance.

#### Future Work

To validate the utility of longitudinal passive data collection, it is essential to demonstrate that errors in sleep quality measures are consistent over time for a given user.

In addition, our study population only includes healthy participants. There is no data on the performance of the device in special populations that might benefit the most from a longitudinal follow-up.

Looking ahead, the contactless devices hold significant potential for a wide range of research and clinical applications [14,64,66-70]. Their capability to monitor sleep in natural environments [68] underscores their versatility and relevance in advancing our understanding of sleep health and its implications.

### Conclusions

In this study, we aimed to (1) assess the performance of a contactless device for sleep stage identification against the gold standard PSG, (2) benchmark it against other comparable devices, and (3) better understand the limitations of such devices. Our paper contributes to those, while also laying out important methodological issues.

This study contributes to the growing body of evidence supporting the utility of nearables for longitudinal sleep monitoring. The WSA’s contactless, undermattress design facilitates unobtrusive data collection in free-living conditions and demonstrates sleep-wake detection performance comparable to established consumer devices. While precise sleep stage classification remains a technical challenge, our results highlight the importance of testing on large, diverse, challenging datasets to capture real-world variability. This research underscores the need for standardized performance assessment methodologies to enable reliable comparisons across devices and studies, offering insights for future device design and guidance for utilization, advancing the goal of improved sleep monitoring for the general population [71].

## Supplementary material

10.2196/77033 Multimedia Appendix 1 Details on methods and algorithm performance per sleep stage.

10.2196/77033 Multimedia Appendix 2 Results of Kruskal-Wallis and Brown-Forsythe statistical tests followed by Holm-Bonferroni correction.

10.2196/77033 Multimedia Appendix 3 Comparison with previous research.

## Abbreviations

AASM: American Academy of Sleep Medicine
ECG: electrocardiogram
EEG: electroencephalogram
EMG: electromyogram
EOG: electrooculogram
MAE: mean absolute error
MCAR: missing completely at random
NEDeep: number of episodes in deep sleep stage
NELight: number of episodes in light sleep stage
NEREM: number of episodes in REM sleep stage
NEWake: number of episodes in wake stage
PIDeep: proportion of the night in deep sleep stage
PILight: proportion of the night in light sleep stage
PIREM: proportion of the night in REM sleep stage
PIWake: proportion of the night in wake stage
PSG: polysomnography
PSQI: Pittsburgh Sleep Quality Index
REM: rapid eye movement (sleep stage)
TIDeep: time spent in deep sleep stage
TILight: time spent in light sleep stage
TIREM: time spent in REM sleep stage
TIWake: time spent in wake stage
TST: total sleep time
WSA: Withings Sleep Analyzer
